# Grafting Tomato ‘Nairouz F_1_’ onto Interspecific Hybrids for Induced Antibiosis and Antixenosis Resistance to *Tetranychus urticae* Koch via Chlorogenic Acid Synthesis

**DOI:** 10.1186/s12870-025-06257-8

**Published:** 2025-03-06

**Authors:** Ahmed M. A. Mahmoud, Ayman H. Mabrouk, Abd-Allah Afifi, Ahmed S. Abdellatif, Neama H. Osman, Mahmoud M. Ahmed

**Affiliations:** 1 Vegetable Crops Department, Faculty of Agriculture, Cairo University, Giza, 12613 Egypt; 2 Zoology and Agricultural Nematology Department, Faculty of Agriculture, Cairo University, Giza, 12613 Egypt; 3 Syngenta Agro S.A.E, Arkan Business Center, El-Sheikh Zayed, 6th October, Giza, Egypt; 4 Genetics Department, Faculty of Agriculture, Cairo University, Giza, 12613 Egypt

**Keywords:** HPLC, Life table parameters, Polyphenols, *Solanum habrochaites*, *S. lycopersicum* L

## Abstract

**Background:**

This study is the first research to investigate the potential of grafting to induce tomato resistance to two-spotted spider mite (TSSM), *Tetranychus urticae* Koch. TSSM can cause up to 50% yield loss of tomato. The grafting technique permits the rapid adoption of biotic/abiotic stress resistance/tolerance from wild relatives as rootstock while preserving the scion’s important horticultural characteristics. Although TSSM resistance is found in wild tomato relatives, particularly those with the *Mi-1* gene, its effects as rootstocks on TSSM resistance remain uncertain. Tomato ‘Nairouz F_1_’ (lacking *Mi-1*) grafted onto six interspecific hybrids with *Solanum habrochiates* (harboring *Mi-1/mi-1*), including commercial rootstock ‘Estamino’ and ‘Fortamino’, along with hybrids between tomato ‘VFN-14’ and each of *S. habrochiates* AusTRCF312064 (R312064), AusTRCF312344 (R312344), CGN15391 (R15391), and LA1777 (R1777). In the 2019 and 2020 fall seasons, the grafted and ungrafted plants were assessed in a naturally TSSM-infested field. The population of TSSM movable stages on grafted and ungrafted plants was estimated one week after transplanting, and every two weeks for 11 weeks. To assess antixenosis and antibiosis resistance in tomato grafts, TSSM males and females were reared on leaves of grafts onto R312064 and R15391, along with ungrafted plants. TSSM bio-behaviors and two-sex life table parameters were assessed.

**Results:**

Grafting onto interspecific hybrids, particularly R15391, R312064, and R312344, significantly lowered TSSM populations compared to commercial rootstocks and ungrafted plants. HPLC analysis revealed that grafting induced foliar synthesis of herbivore-repellent (antixenosis) and antibiosis phenolics, viz., chlorogenic acid in all grafts and syringic acid, pyrocatechol, and vanillin in certain grafts. Grafts-R312064 showed delayed TSSM development, lower survival rates, lower fecundity, and higher mortality. Grafts-R312064 also had a longer mean generation time (*GT*; 23.33 days) and a lower reproductive rate (*R*_*0*_: 14.63), leading to a slower intrinsic population growth rate (*r*_*m*_: 0.115) compared to ungrafted plants and grafts-R15391.

**Conclusion:**

The findings suggest tomato grafting onto rootstocks with the *Mi-1* gene, particularly R312064, could reduce TSSM populations through induced antixenosis and antibiosis resistance mechanisms.

**Supplementary Information:**

The online version contains supplementary material available at 10.1186/s12870-025-06257-8.

## Introduction

Tomato, *Solanum lycopersicum* L., is a popular solanaceous vegetable grown worldwide in fields and greenhouses for nutritional and economic benefits. More than 6.28 million tons were produced in 2022 from 143,618 ha worldwide, averaging 43.70 ton ha^−1^ (http://faostat.fao.org/). Tomato production can be affected by various pests and diseases, which can result in low yields and subpar quality. The two-spotted spider mite (TSSM), *Tetramychus urticae* Koch (Acari: Tetranychidae), is a major tomato pest. TSSM has a wide geographical range, a short life cycle, high offspring production, and a remarkable ability to develop pesticide resistance [1]. TSSM is a polyphagous pest that attacks and feeds on diverse plant species, including over 140 plant families [1]. TSSM feeding causes mesophyll cells to collapse, resulting in tiny white chlorotic spots on leaves caused by chlorophyll degradation. As the feeding damage progresses, the leaves will become yellow or gray and collapse. A severe early TSSM infestation might cause the plant to wilt and die. TSSM feeds on fruits simultaneously, causing yellow or golden spots to emerge on the fruit's surface as it ripens, lowering its market value. TSSM yield losses might be up to 50%, depending on the environment and management strategies [2].

Controlling TSSM is tedious, expensive, and challenging. Host plant resistance, agricultural practices, biological control, and chemical control are the main strategies for sustainable agriculture’s integrated pest management (IPM). Chemical acaricides are effective in reducing TSSM damage, but they pose threats to both the environment and human health, and TSSM quickly develops resistance [1]. Biological control is a vital part of IPM, but it can be inconclusive, particularly in field conditions. The success of biological control depends on several factors, like habitat, soil conditions, and farmers with limited resources are often hesitant to use it [2, 3]. TSSM-resistant cultivars are the simplest, safest, most practical, and ecologically friendly approach to controlling TSSM, reducing its spread, and minimizing yield losses. Cultivated tomato cultivars and lines are susceptible to spider mites [3]. Resistance to arthropods, including TSSM, has been identified in wild species *S. cheesmaniae* [4], *S. galapagense* [5], *S. habrochaites f. hirsutum* [6], *S. habrochaites* f. *glabratum* [7], *S. pennellii* [8], and *S. pimpinellifolium* [9]. *Solanum* sp. exhibits antixenosis and antibiosis resistance mechanisms to TSSM [7–10]. Antixenosis or insect non-preference is a plant property that makes it unattractive for oviposition, feeding, and shelter. Antibiosis refers to the detrimental effects of a plant on the survival, development, or reproduction of insects [9, 10]. Resistance mechanisms have been linked to glandular trichomes and their secretion of allelochemicals such as hydrocarbons and terpenes [9, 11, 12]. Dobzhansky-Muller interactions and other crossing barriers may restrict the breeder’s ability to use resistant wild tomato species and successfully introgression resistance genes into cultivated species [13]. An alternative to breeding and biotechnology techniques for pest resistance is grafting, a surgical method of fusing a scion and a rootstock of two different genotypes with the desired traits. The grafting technique allows the rapid adoption of important economic traits from wild relatives as rootstocks while preserving the scion’s important horticultural traits [14, 15].

Tomato grafting began commercially in the early 1960s as an alternative to methyl bromide to manage soilborne diseases such as fusarium wilt (*Fusarium oxysporum* Schlechtend) and root-knot nematode (*Meloidogyne* spp.). Grafting has considerably more motivations now. Grafting has been widely used to improve yield, fruit quality, and stress management [16]. The effects of grafting on foliar pest populations, such as TSSM, are not fully understood. Few previous studies have reported the effect of grafting on insect pests. Edelstein et al. [17] reported that *Cucurbita maxima* resistance to *T. cinnabarinus* (Acari: Tetranychidae) was acquired by grafting onto *Lignaria siceraria*, but not by watermelon grafting. Rahman et al. [18] found that eggplant grafts onto *S. torvum* had the lowest number of brinjal shoot and fruit borer (*Leucinodes orbonalis* Guenee; Lepidoptera: Crambidae). In grafting eggplant 'A338' onto *S. torvum* 'STT3', Ismail and Hussein [19] reported a decrease in TSSM density (40.11% fewer eggs, 31.71% fewer nymphs, and 27.54% fewer adults) compared to ungrafted plants. Pelletier and Clark [20] found that reciprocal grafting between potato and six wild *Solanum* species could reduce or prevent the Colorado potato beetle (*Leptinotarsa decemlineata* Say; Coleoptera: Chrysomelidae) from attacking scions. Alam et al. [21] reported that tomato grafts onto wild *Solanum* sp. had a lower population of whiteflies (*Bemisia tabaci* Genn.; Hemiptera: Aleyrodidae) compared to ungrafted plants. Alvarez-Hernandez et al. [22] found that tomato grafting onto six *S. lycopersicum* var. *cerasiforme* accessions reduced the populations of *B. tabaci*, potato psyllid (*Bactericera cockerelli* Sulc; Hemiptera: Triozidae), and aphids (*Aphis gossypii* Glover; Homoptera: aphididae). Only two rootstocks slightly impacted the potato aphid (*Macrosiphum euphorbiae* Thomas; Hemiptera: Aphididae). Mandušić et al. [23] found that tomato grafting onto commercial rootstocks ‘Arnold’, ‘Buffon’, ‘Emperador’ and ‘Maxifort’ decreased adult and nymphal populations of *Trialeurodes vaporariorum* Westwood (Hemiptera: Aleyrodidae). According to Žanić et al. [24], tomato grafting onto commercial rootstocks ‘Arnold’, ‘Buffon’, ‘Emperador’, and ‘Maxifort’ reduced the population of *B. tabaci* adults and nymphs on the scion. To understand the grafting mechanism in insect resistance, Žanić et al. [25] found that graft leaves had thinner laminae and mesophyll, and graft phloem sap exudates had more leucine and lysine, making grafts less attractive to *B. tabaci*. Ismail and Hussein [19] found that eggplant grafts onto *S. torvum* had higher activity of superoxide dismutase and catalase, as well as higher foliar content of photosynthetic pigments, compared to non-grafted plants.

The most compatible and often used tomato rootstocks are intra/interspecific hybrids [15, 26]. Interspecific hybrids are more vigorous and frequently produce high-quality rootstocks, increasing the rootstock’s genetic diversity [15, 27]. Several F_1_ *S. lycopersicum* × *S. habrochaites* hybrid rootstocks are commercially available with various resistances to soilborne diseases. Several interspecific F_1_ hybrids harbor the *Mi-1* gene, e.g., ‘Maxifor’, ‘Beaufort’, and ‘Emperador’ [28]. *S. habrochaites* accessions provide high genetic diversity for resistance/tolerance traits to biotic/abiotic stresses [29], and some of its accessions have shown high resistance to arthropods, including TSSM [6, 7]. Antibiosis and antixenosis resistance mechanisms of tomato to TSSM were identified, mediated by trichomes, particularly glandular ones, and their secretions [10]. The *Mi-1* gene is a dominant gene that confers resistance to parasitized phloem tissue culture. *Mi-1* was first identified in wild relative *S. peruvianum* as a resistance gene to three root-knot nematode species: *M. incognita* (Kofoid & White) Chitwood, *M. javanica* (Treub) Chitwood, and *M. arenaria* (Neal) Chitwood. *Mi-1* gene also mediated resistance against three arthropod species: *M. euphorbiae* [30], *B. tabaci* biotype B and Q [31], and *B. cockerelli* [32]. Aphid resistance first appears in fully expanded leaves of four- to five-week-old tomato plants [30]. *Mi-1*-mediated resistance has antibiotic effects, with 100% mortality occurring within 10 days on resistant plants [33]. According to Martinez de Ilarduya et al. [34], *Mi-1*-mediated resistance to the potato aphid is developmentally controlled and does not involve hypersensitive resistance. *Mi-1*-RNA is present throughout development and is not induced by herbivory, indicating that *Mi*-mediated resistance is regulated at the translational or posttranslational levels. According to Godzina et al. [35] (2010), tomato ‘Mottelle’ (*Mi-1*/*Mi-1*) had no significant influence on TSSM’s reproductive capacity and could not be a reliable source of TSSM resistance. Keskin and Kumral [36] found that the commercial rootstock ‘Beaufort’ had a significantly lower TSSM population than those on tomato cultivars. Studies Žanić et al. [24] and Mandušić et al. [23] found that these rootstocks are efficient against *B. tabaci* and *T. vaporariorum*.

This study evaluated the efficacy of grafting tomato ‘Nairouz F_1_’ onto interspecific hybrids with *S. habrochaites*, harboring the *Mi-1* gene, for TSSM resistance compared to ungrafted plants under natural field infestation conditions. High-performance liquid chromatography (HPLC) analysis for leaf phenolic compounds was used to assess how grafting influences TSSM resistance. Furthermore, TSSM life table parameters were estimated on some grafted and ungrafted plants to understand their impact on development, survival, and reproduction of TSSM population and predicate future demographic changes in the TSSM population.

## Materials and Methods

### Plant materials

The rootstocks consisted of four tomato interspecific hybrids and two commercial rootstocks (Table 1). Interspecific hybrids were crossed between a female parent, *S. lycopersicum* LA815 'VFN-14', which harbors the *Mi-1*/*Mi-1* gene, and each of the male parents, *S. habrochaites* accessions AusTRCF312064, AusTRCF312344, CGN15391, or LA1777 (Table 1). Tomato ‘Nairouz F_1_’ is used as a scion (Table 1). The grafted and ungrafted plants were evaluated in a naturally TSSM-infested field at the Faculty of Agriculture, Cairo University, Giza, Egypt (30°01′05.6″N 31°12′24.3″E) during the 2019 and 2020 fall seasons.

**Table 1:** The used plant materials

**Germplasm**			**Code**	***Mi-1 gene***	**Phenotype**
**Interspecific hybrids**	Female parent	*Solanum lycopersicum* LA0815 cv. VFN-14		*Mi-1*/*Mi-1*	Resistance to *Verticillium alboatrum*, *Fusarium oxysporum* f.sp. *lycopersici*, and *Meloidogyne sp.*
	Male parents	*S. habrochaites* AusTRCF312064	R312064	*Mi-1*/*mi-1*	
		*S. habrochaites* AusTRCF312344	R312344	*Mi-1*/*mi-1*	
		*S. habrochaites* CGN 15391	R15391	*Mi-1*/*mi-1*	
		*S. habrochaites* LA 1777	R1777	*Mi-1*/*mi-1*	
**Commercial rootstocks**	‘Estamino’		Estamino	*Mi-1*/*mi-1*	Highly resistant to Tomato Mosaic Virus (ToMV), *Fulvia fulva*, *Phytophthora infestans*, *V. alboatrum*, *V. dahlia*,* F. oxysporum* f.sp. *lycopersici*, and *F. oxysporum* f.sp. *radices-lycopersici* Intermediated resistant to Tomato Spotted Wilt Virus (TSWV), *Pyrenochaeta lycopersici*,* M. arenaria*, *M. incognita*, and *M. javanica*
	‘Fortamino’		Fortamino	*Mi-1*/*mi-1*	Highly resistant to ToMV, *F. fulva*, *P. infestans*, *V. alboatrum*, *V. dahlia*, and *F. oxysporum* f.sp. *lycopersici* Resistance to TSWV, *M. arenaria*, *M. incognita*, and *M. javanica*
**Scion**	*S.lycopersicum* cv. TH99806 (Nairouz F_1_)			*mi-1*/*mi-1*	Semi-indeterminate plant, high-resistance to Fusarium (1&2); ToMV (0–2), and Verticillium Intermediated resistance: TYLCV

Accession the AusTRCFs were gifted by the Australian Tropical Crops & Forages Genetic Resources Center, Queensland, Australia (https://www.2.dpi.qld.gov.au); the CGN was gifted by the Center for Genetic Resources, Wageningen University, the Netherlands (http://cgngenis.wur.nl); and the Las were gifted by the University of California, Davis, USA (http://www.tgrc.ucdavis.edu). Commercial rootstocks were purchased from Enza Zaden Company (https://www.enzazaden.com/). Scion was purchased from Syngenta Company, Egypt (https://www.syngenta.com.eg)

### Detection of the *Mi-1* gene in plant materials

The *Mi-1* gene in tomato genotypes was detected using three PCR-based markers, *Mi23*-SCAR (sequence characterized amplified region; F: TGGAAAAATGTTGAATTTCTTTTG and R: GCATACTATATGGCTTGTTTACCC) [37]. PMi12-SCAR (F; CCTGCTCGTTTACCATTACTTTTCCAACC and R: CTGCTCGTTTACCATTACTTTTCCAACC) [38], and *Rex-1*-CAPS (cleaved amplified polymorphic sequence; F: TCGGAGCCTTGGTCTGAATT and R: GCCAGAGATGATTCGTGAGA) [39]. Tomato ‘VFN-14’ was used as a positive control for the *Mi-1/Mi-1* gene.

The CTAB technique [40] was used to isolate DNA from healthy fresh leaves of rootstocks and scion seedlings. Total genomic DNA was quantified using a UV/Vis spectrophotometer at 260 nm (OD_160/280_ = 1.8–2.0) and adjusted to about 10 ng μL^−1^ concentration. The PCR amplification was performed in a total volume of 25 μL containing 5 μL 5 × PCR buffer, 2.5 μL 2.5 mM dNTP, 2.5 μL 2.5 mM MgCl_2_, 2–5 μL of DNA extract, 0.1 μL unit Taq DNA polymerase (Vivantis, Selangor DE, Malaysia), 2.5 μL 10 μM of each forward and reverse primer, and sufficient ddH_2_O to the final volume. PCR amplification was performed using a thermocycler (Eppendorf® Mastercycler Gradient 5, Hamburg, Germany). The 35-cycle PCR reaction was performed as follows: 30s of denaturation at 94 °C, 30s of annealing at 52 °C (Rex-1 and PMi12) and 56 °C (Mi23), 1 min of polymerization at 72 °C, and 5 min of final extension at 72 °C. Primarily, 5 ml of each primer reaction was loaded onto a 1.5% agarose gel to ascertain whether PCR amplification was successful. No restriction enzymes digested the Mi23 and PMi12 PCR products. Taq-1 was used to digest 10 ml of Rex-1 PCR product. PCR products were resolved in 1.5% agarose gel in 1 × tris–acetate-EDTA buffer. DNA bands were stained with ethidium bromide (0.5 μg mL^−^1) and photographed under UV light using a gel documentation system (Bio-Rad® Gel Doc-2000). The 1 kb ladder DNA was used as the molecular weight size marker.

### Procedures for grafting and experimenting

On July 1st of both seasons, the scion and rootstock seeds were sown in 209- and 150-cell seedling trays, respectively. The trays were filled with a 1:1 volume ratio of peat and vermiculate enriched with macro- and microelements. After 7–10 days of sowing, seedling trays were placed in a greenhouse at 26 ± 2°C. Daily, seedlings were fertigated with a commercial fertilizer solution (20:20:20, N: P: K; 1g L^−1^). After 20–25 days of sowing, seedlings with three to four true leaves were grafted using the slant-cut technique. The grafts were immediately placed in a clear, closed, and shady plastic growth chamber at 28 ± 2° C and above 95% relative humidity for three days. Starting on the third day, the relative humidity in the growth chamber steadily decreased as the amount of light increased. The grafts were transferred to an acclimatization greenhouse after 7 days of grafting. Grafts and un-grafted plants were transplanted 50 days after sowing.

The grafted and ungrafted transplants were transplanted at the acarology greenhouse in mid-August. The greenhouse was covered with black saran fabric with narrow holes to keep insects out of the greenhouse. The transplants were transplanted into beds (1.2 m width × 14 m long) with a 30 cm spacing between transplants under a drip irrigation system. A randomized complete block design (RCBD) was used to arrange the grafted and ungrafted plants in three replications. Each experimental unit (EU) consisted of 10 plants. Fertilization, irrigation, and weeding were performed according to agricultural practices for commercial tomato production without the use of insecticides. 105.8 N: 24.9 P_2_O_5_: 88.1 K_2_O: 10 CaO kg ha^−1^ was used to fertigate tomato plants during the growing season^15^.

### Source of TSSM infestation

**Fig. 1:**
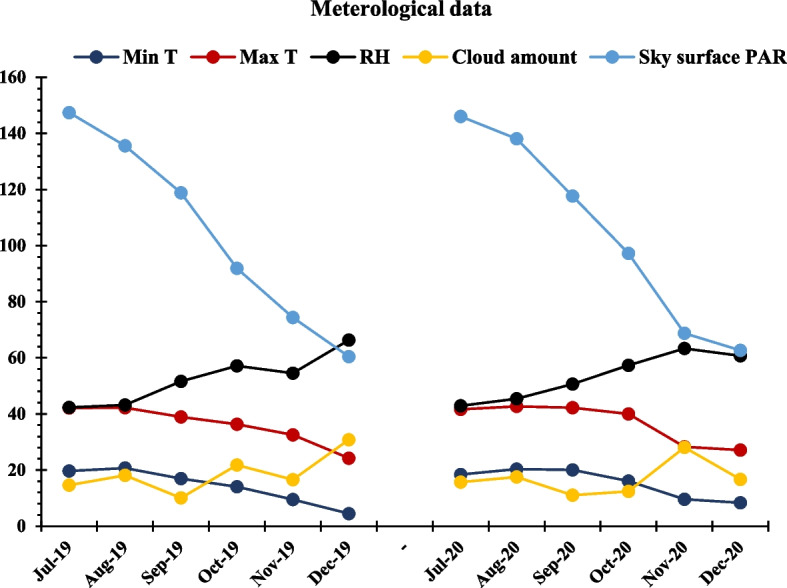
Meteorological data during the 2018 and 2019 fall seasons. Meteorological data are presented as the average monthly readings of the maximum (Max T: °C) and minimum (Min T: °C) temperatures, relative humidity (RH: %), cloud amount (%), and sky surface photosynthetically active radiation (Sky surface PAR: W m^−2^) during the fall 2018 and 2019 seasons (https://power.larc.nasa.gov/data-access-viewer/)

The TSSM infestation was caused by a native mite population on wild weed plants growing in the field. Three days after transplanting, the infested weed plants were uprooted and distributed throughout the field to allow the movable stages of TSSM to transfer to tomato plants. Tomato plants were infested by TSSM during the first week after transplanting. TSSM grew and spread during the experiment due to the suitable climatic conditions (Fig. 1).

### *Estimation of the movable stages of TSSM* p*opulation*

The population of TSSM movable stages on grafted and ungrafted plants was estimated one week after transplanting, and then every two weeks for 11 weeks in both seasons. In the early morning, 30 leaves were collected from the upper half of the plant for each treatment at each plant age (ten leaves / EU). The leaf samples were randomly collected from plants according to the RCBD-factorial design, with two factors: grafting/rootstocks (the first factor) and plant age (the second factor). The collected leaves were put in a polyethylene bag with wet tissue paper, sealed with rubber bands, and put in an icebox to keep them fresh until transport to the Acarology Lab. The movable TSSM stages per leaflet were counted using a stereomicroscope (SD30, Olympus, Japan).

### TSSM graft resistance mechanisms

The foliar polyphenolic components of grafted and ungrafted plants were estimated using high-performance liquid chromatography (HPLC) as a chemical feature associated with TSSM resistance. Furthermore, the biological behaviors of TSSM on grafts onto R-312064 and R-1777 and ungrafted plants were investigated by estimating life table parameters to gain a deeper understanding of their development, survival, reproduction, and future population changes.

#### Qualitative determination of polyphenols using HPLC analysis

Fresh fully expanded fresh leaves from the upper third of both grafted and ungrafted plants were collected in the early morning three months after transplanting. The collected leaves were put in polyethylene bags, sealed with a rubber band, and placed in an icebox for transportation to the Chromatography Laboratory, Central Laboratories Network, National Research Centre, Giza, Egypt. According to Matilla et al. [41], HPLC was used to determine polyphenolic compounds in the foliar extracts. Sixteen phenolic and flavonoid compounds, including gallic acid, chlorogenic acid, catechin, methyl gallate, caffeic acid, syringic acid, pyrocatechol, rutin, ellagic acid, coumaric acid, vanillin, ferulic acid, naringenin, taxifolin, cinnamic acid, and kaempferol, were dissolved in methanol to be used as standards. The polyphenolic compounds were determined by an Agilent Technologies 1260 series HPLC system (Agilent, USA). The Kromasil C18 column (4.6 mm × 250 mm, 5 μm, Labio, Czech Republic) was used to separate the material. The mobile phase consisted of water (A) and 0.05% trifluoroacetic acid in acetonitrile (B) at a flow rate of 1ml min^−1^. In a linear gradient, the mobile phase was set to 0 min (82% A), 0–5 min (80% A), 5–8 min (60% A), 8–12 min (60% A), 12–15 min (85% A), and 15–16 min (82% A). A multiwavelength detector was inspected at 280 nm. The injection volume for each sample solution was 10 μl. The column temperature was maintained at 35 °C. The peak in HPLC was determined by comparing the retention time of the reference standards. Peaks were identified by comparing congruent retention times and UV spectra with those of the standards [42].

### TSSM biological behavior

#### TSSM rearing

A TSSM colony was collected from the field’s infected tomato plants. The colony was reared in the acarology lab using leaf discs (3cm diameter) from copper acalypha shrubs (*Acalypha wilkesiana* Müll. Arg.) to provide a consistent supply of mites for biological behavior evaluation. The acalypha leaf discs were put on wet cotton pads in Petri dishes (9 mm). A 1 cm wide strip of absorbent cotton was placed around the leaf disc’s edge to prevent the mites from escaping. Dishes were incubated at 27 ± 3 °C, 65 ± 5% RH, and L16:D8 h photoperiods. The cotton pads were wetted daily. The leaves were replaced every four days, and mites were gently brushed onto the new leaves.

#### No-choice assay

The biological behavior of TSSM was studied on grafts onto either R-312064 or R-1777 and ungrafted plants, which differed in the population of TSSM moveable stages (larva, protonymph, deutonymph, and adult). A no-choice assay was performed in separate foam dishes (13.5 × 17.5 cm) with leaf discs (3 cm diameter; 10 discs/dish) on wet cotton pads under laboratory conditions at 27 ± 3 °C, 65 ± 5% RH, and L16:D8 h photoperiods. The leaf discs were made from the upper third of the plant's 90 DAT. The assay involved wetting cotton pads daily and replacing leaf discs every three days. Three no-choice assays (one dish/treatment) were conducted using a randomized complete design (CRD).

The initial step of the assay was to collect mated females with enlarged belly ends from the rearing and put them into leaf discs (female/disc) for oviposition. After 24h, the female mites were removed and the number of oviposited eggs was counted. The deposited eggs per leaf disc were observed daily until they hatched into larvae. The newly hatched larvae were left to feed, and their developmental stages were estimated until adulthood. New adults were counted and sorted by gender. According to Alford [43], females are characterized by an intense red color and a length of about 0.46 mm, while males are characterized by a yellow-green color, thinner backs, and a length of about 0.25 mm. A pair of adults (a male and a female) were transferred onto fresh leaf discs (one pair per disc). New males were added to the leaf discs when males died before females. The analysis did not include dead males. During the assay, leaf discs were changed daily, and the number of mite eggs on the removed leaf discs was counted. Eggs were observed daily to determine the days of hatching (eggs to larvae), offspring mortality during the life cycle, longevity, and female fecundity (eggs per female). Assessments were performed until the last female died.

According to Chi et al. [44], the life table parameters of TSSM fed on the leaves of grafted and ungrafted plants were estimated using data from all tested male and female individuals, including those who died during the immature stage. Table S1 displays the equations for two-sex life table parameters.

Life table parameters can provide researchers with a variety of data, including the age-stage distribution of all individuals over time. The age-stage distribution describes the duration of the biological stages that compose the insect’s life cycle, usually expressed in days. Such information is particularly useful for assessing the bioecology of insect species and developing mathematical models that describe their biology [44].

### Statistical analysis

The data collection of the TSSM movable stages population was initially tested for normality using the Shapiro–Wilk test. An ANOVA was performed according to a RCBD for a factorial scheme that involved six plant ages and seven grafting treatments [45]. Tukey’s multiple range test was used to compare significant means with a 5% confidence level [45]. ANOVA and mean comparisons were performed using MSTAT-C v.2.1 (Michigan State University, Michigan, USA).

The life table parameters associated with the TSSM population were estimated using the bootstrap procedure with 100,000 re-sampling using TWOSEXMS-Chart software (< http://140.120.197.173/Ecology/prod02.htm >) according to Wei et al. [46]. A paired bootstrap test was used to compare the means [46].

## Results

### The presence of the *Mi-1* gene in plant materials

**Fig. 2:**
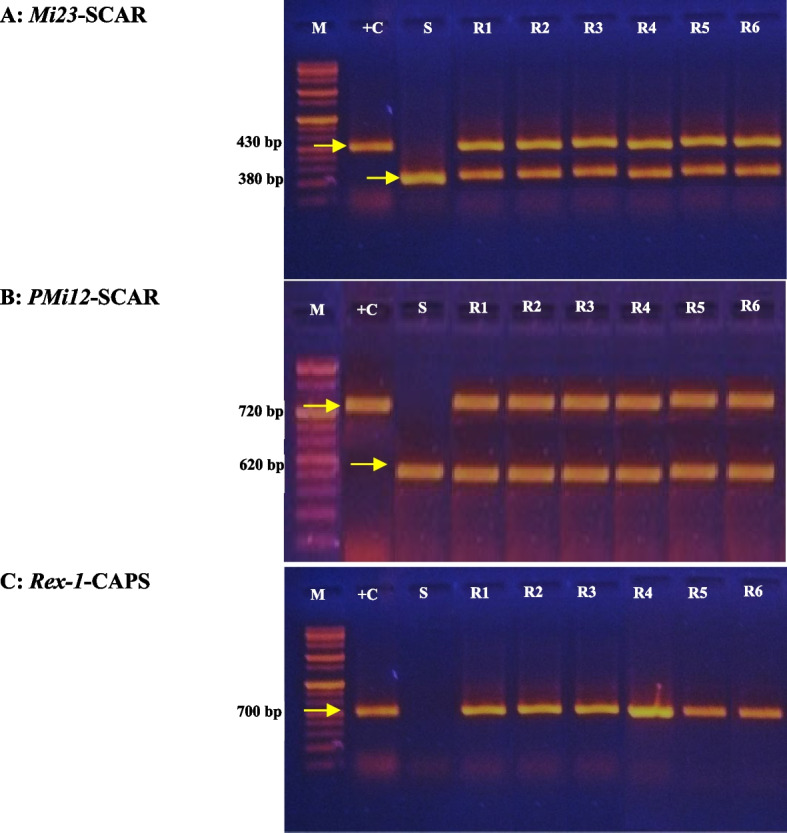
Detection of the *Mi-1* gene by PCR-based markers *Mi-23* (**A**), *PMi12* (**B**), and *Rex-1* (**C**) in tomato rootstocks and scion. Lanes M: 1kbp DNA marker; + C: *S. lycopersicum* LA815 ‘VFN-14’ (harboring *Mi-1*/*Mi-1*); S: scion *S. lycopersicum* ‘Nairouz F_1_’; R1-R4: interspecific hybrids between *S. lycopersicum* ‘VFN-14’, as a female parent, and each of *S. habrochaites* AusTRCF312064 (R1), AusTRCF312344 (R2), CGN15391 (R3), and LA1777 (R4), as male parents; and R5:R6: commercial rootstocks ‘Estamino’ and ‘Fortamino’

Detection of the *Mi-1* gene in tomato germplasm is shown in Fig. 2, using PCR-based markers *Mi23*, *PMi12*, and *Rex-1*. A single 380 bp fragment for the homozygous genotype (*Mi*−1/*Mi*−1) was obtained with the cultivar VFN-14 using the *Mi23* marker (Fig. 2A). A 430 bp fragment was obtained with plants without the *Mi-1* gene (*mi-1/mi-1*), as shown in Fig. 2A for tomato scion ‘Nairouz F_1_’. Two fragments, 380 and 430 bp, were produced by heterozygous genotypes (*Mi*−1/*mi*−1) as observed in rootstocks (Fig. 2A). PCR with the *PMi12* primer produced a single 720 bp band for the homozygous resistant (*Mi-1*/*Mi-1*) cultivar VFN-14, and a single 620 bp band for the homozygous susceptible (*mi-1*/*mi-1*) cultivar Nairouz F_1_ (Fig. 2B). Heterozygous germplasm (*Mi-1*/*mi-1*) yielded two fragments, 720 and 620bp, as observed with all rootstocks (Fig. 2B). The PCR-*Rex-1* marker generated about a 720 bp band for all tomato genotypes (Fig. 2C). *Taq-1* digestion of PCR products produced 560 and 160 bp fragments in homozygous resistant plants (*Mi-1*/*Mi-1*), as found with ‘VFN-14’. The heterozygous germplasm (*Mi-1*/*mi-1*) yielded three bands, 720, 560, and 160 bp, as rootstocks and the scion (Fig. 2C).

### The population of TSSM movable stages

Table 2 presented that rootstocks (R), plant age (PA), and the interaction R × PA had highly significant (*P* < 0.001) effects on the population of TSSM movable stages (leaflet^−1^) during both seasons. During both seasons, PA had the largest incidence of total variance (SS%) (90.6 and 94.1%, respectively), followed by each of R (4.6 and 2.5%, respectively) and the interaction R × PA (4.4 and 2.7%, respectively).

**Table 2:** Population (leaflet^-1^) of *Tetranychus urticae* moveable stages on grafted and ungrafted tomato ‘Nariouz F_1_^’^ during the 2019 and 2020 fall seasons

**Rootstock**^y^	**Plant age (week)**^z^											
	**1**		**3**		**5**		**7**		**9**		**11**	
**Fall 2019**												
** Ungrafting**	0.90±0.15	q-t	2.83±0.19	n-p	5.17±0.26	k-m	7.93±0.15	hi	12.95±0.13	cd	18.40±0.25	a
** R312064**	0.73±0.09	q-t	1.90±0.15	p-s	3.50±0.12	n	6.13±0.18	jk	8.87±0.29	gh	11.78±0.35	de
** R312344**	0.50±0.06	t	2.07±0.12	o-q	5.13±0.19	k-m	8.33±0.41	hi	10.90±0.17	ef	15.70±0.45	b
** R15391**	0.63±0.07	r-t	1.50±0.17	p-t	3.30±0.23	no	5.57±0.12	kl	7.87±0.30	hi	10.23±0.20	fg
** R1777**	0.63±0.12	r-t	1.97±0.12	o-r	4.20±0.12	l-n	7.27±0.23	ij	11.63±0.38	de	15.17±0.19	b
** ‘Estamino’**	0.70±0.06	q-t	1.87±0.15	p-t	5.37±0.12	k-m	8.42±0.12	hi	13.27±0.19	c	18.27±0.29	a
** ‘Fortamino’**	0.53±0.09	st	1.60±0.06	p-t	4.17±0.19	mn	8.50±0.68	hi	14.73±0.44	b	17.57±0.32	a
**Fall 2020**												
** Ungrafting**	0.53±0.09	pq	1.13±0.12	n-q	3.37±0.15	j	5.80±0.12	gh	7.87±0.18	d	11.60±0.23	a
** R312064**	0.60±0.06	pq	1.00±0.15	o-q	2.53±0.15	j-l	4.67±0.12	i	6.30±0.44	fg	8.80±0.36	c
** R312344**	0.50±0.06	pq	1.17±0.09	n-q	2.50±0.21	j-l	4.50±0.15	i	6.80±0.45	ef	9.73±0.18	b
** R15391**	0.67±0.03	pq	1.33±0.07	m-p	2.17±0.15	k-m	2.90±0.12	jk	4.93±0.26	hi	7.00±0.12	d-f
** R1777**	0.40±0.06	q	1.13±0.09	n-q	1.83±0.09	l-o	4.60±0.23	i	7.33±0.18	de	9.63±0.12	bc
** ‘Estamino’**	0.53±0.03	pq	1.30±0.15	m-p	2.27±0.09	kl	4.77±0.32	i	7.40±0.15	de	10.07±0.12	b
** ‘Fortamino’**	0.60±0.06	pq	1.37±0.15	m-p	2.00±0.06	l-n	4.30±0.17	i	6.20±0.31	fg	9.60±0.17	bc
			**2019**				**2020**					
**Source of variance**	**df**^x^		**SS**^x^ (%)		**MS**^x,w^		**SS**^x^ (%)		**MS**^x,w^			
** Replication**	**2**		**0.04**		0.754^*^		**0.23**		1.549^***^			
** Rootstock (R)**	**6**		**4.63**		28.785^***^		**2.54**		5.647^***^			
** Plant age (PA)**	**5**		**90.63**		677.092^***^		**94.10**		251.498^***^			
** R × PA**	**30**		**4.35**		5.415^***^		**2.72**		1.211^***^			
** Error**	**82**		**0.35**		**0.160**		**0.41**		**0.067**			

^z^Means value ± standard error (n=4). Means followed by the same letters in each season are not significantly different according to Tukey’s multiple range test (*p <0.05*).

^y^Rootstocks were tomato interspecific hybrids between *Solanum lycopersicum *LA815 ‘VFN-14’, as a female parent and each of *S. habrochaites *AusTRCF312064 (R312064), AusTRCF312344 (R312344), CGN15391 (R15391), and LA1777 (R1777) as male parents; and commercial rootstocks ‘Estamino (R5) and ‘Fortamino’.

^x^df is degrees of freedom, SS is a variance as a ratio of the total variance, and MS is a mean of squares.

^w*^, ^**^, and ^***^Significant at 5 and 0.1% level of probability, respectively.

**Fig. 3:**
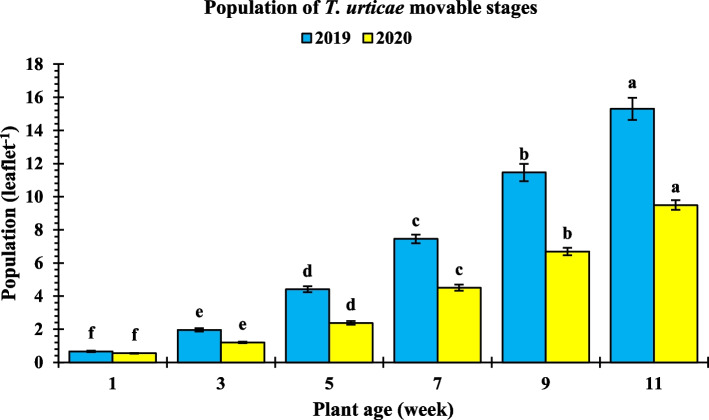
The population of *Tetranychus urticae* movable stages on tomato ‘Nariouz F_1_’ at different ages during the 2019 and 2020 fall seasons. The population was averaged for the grafted and un-grafted plants. Columns for each season with the same letter represent no significant difference according to Tukey's multiple range test (*P* < 0.05). Vertical bars represent ± standard error of the mean (*n* = 21)

The TSSM population until week 3 after transplanting on tomato ‘Nairouz F_1_’ grafted and ungrafted plants was within the economically safe range (< 1 mite leaflet^−1^) for both seasons, as presented in Fig. 3. Figure 3 illustrates that the population of TSSM moveable stages gradually grew as plants became older, including both grafted and ungrafted plants. During both seasons, there was a significant (*P* < 0.05) increase in population at the oldest age, 11 weeks (15.30 and 9.49, respectively), and a significant (*P* < 0.05) decrease at the youngest age, 1 week (0.66 and 0.55, respectively) (Fig. 3). The plant’s leaf area increases as it ages, as does the vegetative surface area that can be infested.

**Fig. 4:**
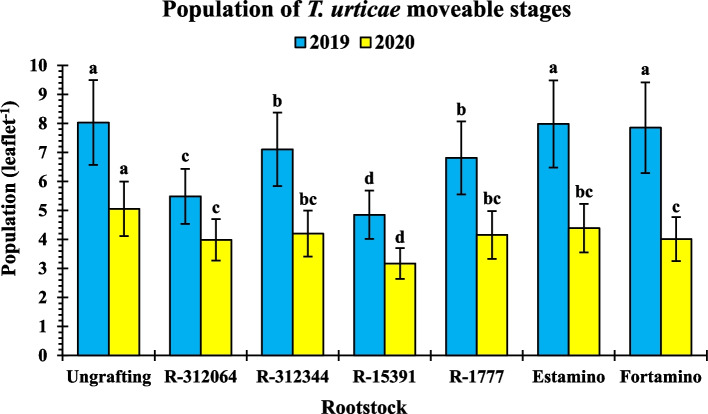
The population of *Tetranychus urticae* movable stages on grafted and ungrafted tomato ‘Nariouz F_1_’ during the 2019 and 2020 fall seasons. The population was averaged for 11 weeks. Rootstocks are presented in Table 1. Columns for each season with the same letter represent no significant difference according to Tukey's multiple range test (*P* < 0.05). Vertical bars represent ± standard error of the mean (*n* = 18)

The population of TSSM moveable stages was significantly (*P* < 0.05) lower in the grafts onto interspecific hybrids compared to those onto commercial rootstocks and ungrafted plants during both seasons and an average of 11 weeks of plant age, as shown in Fig. 4. Grafts-R15391 had significantly lower (*P* < 0.05) TSSM populations during both seasons (4.85 and 3.17, respectively), followed by grafts onto R312064 (5.49 and 3.98, respectively), R312344 (7.11 and 4.20, respectively), and R1777 (6.81 and 4.16, respectively). Ungrafted plants had a significantly larger (*P* < 0.05) TSSM population during both seasons (8.03 and 5.05, respectively), with no significant (*P* < 0.05) differences than grafts onto commercial rootstocks in the first season (Fig. 4).

Table 2 revealed that grafts onto interspecific hybrids had a significantly similar (*P* < 0.05) TSSM population to ungrafted plants in the early plant ages, i.e., 1–5 weeks, during both seasons. Significant differences (*P* < 0.05) appeared between the grafted and ungrafted plants starting from the seventh week after transplanting, as presented in Table 2. Ungrafted plants had the highest significant (*P* < 0.05) TSSM population at 11 weeks after transplanting in both seasons (18.40 and 11.60 mite leaflet^−1^, respectively). Still, there were no significant differences (*P* < 0.05) among ungrafted plants and grafts onto ‘Estamino’ and ‘Fortamino’ at the same age in the first season only (18.27 and 17.57 mite leaflet^−1^, respectively; Table 2). During the seventh to eleventh weeks after transplanting, the TSSM population on grafts-R15391 was significantly lower (Table 2).

### Plant metabolism of phenols

**Fig. 5:**
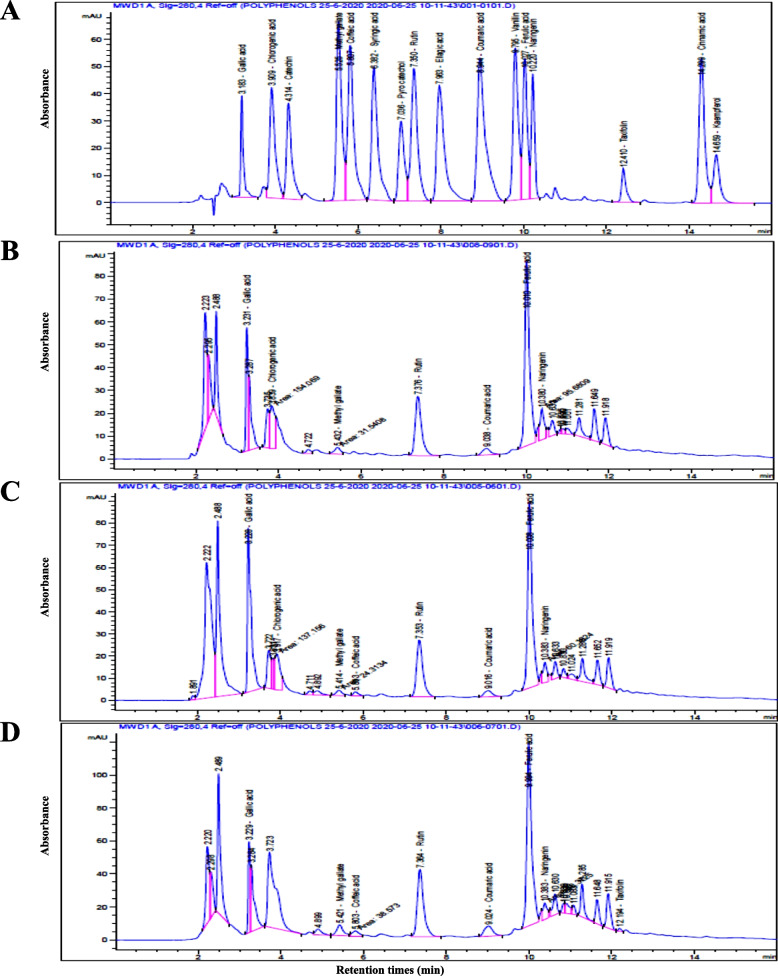
HPLC chromatogram of polyphenols in a standard solution (**A**) and foliar extracts from graft-R312064 (**B**), graft-R15391 (**C**), and ungrafted plants (**D**) of tomato ‘Nairouz F_1_’. Rootstocks were interspecific hybrids between *Solanum lycopersicum* LA0815 cv. VFN-14, as a female parent, and either of *S. habrochaites* AusTRCF312064 (R-312064) or CGN15391 (R-15391), as male parents. Peaks of a standard solution (A) were 1: gallic acid, 2: chlorogenic acid, 3: catechin, 4: methyl gallate, 5: coeffeic acid, 6: syringic acid, 7: pyrocatechol, 8: rutin, 9: ellagic acid, 10: coumaric acid < 11: vanillin, 12: ferulic aicd, 13: naringenin, 14: taxifolin, 15: cinnamic acid, and 16: kaempferol

The polyphenols in leaf extracts of ‘Nairouz F_1_’ grafted and ungrafted plants grown under TSSM infestation were identified using HPLC with 16 different polyphenol standards. Table 3 and Fig. 5A show that the reaction time (RT) for standard phenols greatly varied between 3.18 min for gallic acid to 14.66 min for kaempferol. The qualitative analysis was contingent on the RT provided by various polyphenols, as demonstrated in Fig. 5 and Table 3. According to Table 3, tomato leaf extracts contained 12 out of 16 phenolic compounds. The tomato leaf extracts do not contain catechin, ellagic acid, kaempferol, and cinnamic acid (Table 3). Ungrafted plants revealed eight phenolic compounds with peak ratios that ranged from 0.23% for taxifolin to 22.35% for ferulic acid (Fig. 5D). Tomato grafts revealed a range of 7 compounds for those onto R-312064 to 12 compounds for those onto R-1777. The peak ratios of phenolic compounds in grafted plants were higher (from 47.29% with grafts onto R-15391 to 55.49% with grafts onto R-312064) than in ungrafted plants (44.65%). Grafts onto R1777 had the most phenolic compounds (12 compounds with peak ratios ranging between 0.38–22.23%), followed by those onto both R32344 and ‘Estamino’ (10 compounds for both with peak ratios ranging between 0.36–17.42% and 0.36–14.51%, respectively).

**Table 3:** Polyphenolic profiles in grafted and ungrafted tomato ‘Nariouz F_1_^’^ grown under *Tetranychus urticae *Koch. infestation

**Polyphenol standard solution at 280 nm**				**Name**	Peak area (%) of polyphenol compounds in grafts leaves on different rootstocks^x^						
**Peak**	RT^z^(min)	Area^y^(mAU*s)	Area^y^%		**Estamino**	**Fortamino**	**R312344**	**R1777**	**R312064**	**R15391**	**Ungrafting**
1	3.18	169.2	2.56	Gallic acid	14.57	9.95	7.82	4.78	7.56	13.55	4.79
2	3.91	380.91	5.76	Chlorogenic acid	7.00	4.86	14.42	5.07	5.92	3.91	ND
3	4.31	321.14	4.85	Catechin	ND	ND	ND	ND	ND	ND	ND
4	5.53	577.6	8.73	Methyl gallate	0.91	1.13	0.7	1.3	1.21	0.69	1.77
5	5.81	593.04	8.96	Coffeic acid	0.36	0.55	0.36	0.93	ND	0.45	0.95
6	6.38	516.51	7.81	Syringic acid	0.53	ND	1.03	0.4	ND	ND	ND
7	7.04	289.06	4.37	Pyro catechol	ND	ND	ND	0.31	ND	ND	ND
8	7.35	530.74	8.02	Rutin	11.11	11.79	3.71	9.5	10.66	7.58	10.66
9	7.96	556.84	8.42	Ellagic acid	ND	ND	ND	ND	ND	ND	ND
10	8.94	720.92	10.9	Coumaric acid	1.47	ND	1.13	2.17	1.55	1.09	2.21
11	9.8	495.39	7.49	Vanillin	ND	0.4	ND	0.93	ND	ND	ND
12	10.03	401.78	6.07	Ferulic acid	14.51	22.57	17.21	22.23	24.91	18.7	22.35
13	10.22	262.06	3.96	Naringenin	1.17	1.7	1.42	1.45	3.68	1.71	1.69
14	12.41	95.24	1.44	Taxifolin	1.16	ND	1.27	0.38	ND	ND	0.23
15	14.3	512.57	7.75	Cinnamic acid	ND	ND	ND	ND	ND	ND	ND
16	14.66	192.2	2.91	Kaempferol	ND	ND	ND	ND	ND	ND	ND
Total			100		**52.79**	**52.95**	**49.45**	**49.07**	**55.49**	**47.68**	**44.65**

^z^*RT* Retention time.

^y^Compound expressed as miliabsorbance units × second (mAU×s) and percentage.

^x^Rootsotcks are presented in Table 1.

*ND* not-detected.

Chlorogenic acid was found in all grafts, but not in ungrafted plants (Table 3 and Fig. 5). The peak chlorogenic acid ratios for grafts onto R-312344 or 'Estamino' were higher (14.41 and 7.00%, respectively) than those for other grafts (3.91–5.92%) (Table 3 and Fig. 5). Synringic acid was found in grafts onto 'Estamino', R312344, and R1777, with peak ratios of 0.53, 1.03, and 0.4%, respectively, compared to ungrated plants, as indicated in Table 3. Vanillin was found only in grafts onto both 'Fortamino' and R1777, with peak ratios of 0.4 and 0.93%, respectively (Table 3). Pyro-catechol was only found in grafts onto R1777, with a peak ratio of 0.31% (Table 3).

Both grafted and ungrafted plants contained gallic acid, methyl gallate, rutin, ferulic acid, and naringenin (Table 3). Grafts had higher gallic acid peak ratios (ranging from 7.56 to 14.57%), except for grafts onto R1777 (4.78%), which was similar to ungrafted plants (4.79%). Ungrafted plants had the greatest peak methyl gallate ratio (1.77%) compared to grafts (0.7–1.21%) (Table 3). Ungrafted plants and Grafts onto ‘Fortamino’, ‘Estamino’ exhibited the highest peak rutin ratios (11.79 and 11.11%, respectively), followed by grafts onto R-312064 and ungrafted plants (11.66% for both), and then the other grafts (3.71–9.5%). Grafts onto R-312064 had the highest naringenin ratio (3.68%), followed by those onto R-15391 (1.71%) and ‘Fortamino’ (1.70%), and ungrftaed plants (1.69%), and then the other grafts (1.17–1.45%) (Table 3). The peak ferulic acid ratios were in order as follows: grafts-R312064 (24.91%), grafts- ‘Fortamino’ (22.57%), ungrafted plants (22.35%), grafts-R1777 (22.23%), grafts-R15391 (18.70%), grafts-R312344 (17.21%), and grafts- ‘Estamino’ (14.51%).

Taxifolin was found in ungrafted plants and grafts onto ‘Estamino’, R32344, and R1777. Taxifolin peak ratios ranged from 0.38 to 1.27% in grafts, but were only 0.23% in ungrafted plants (Table 3). All grafts showed reduced peak ratios of methyl gallate (0.7–1.21%), coffeic acid (0.36–0.93%), and coumaric acid (1.09–2.17%) compared to ungrafted plants (1.77, 0.95, and 2.21%, respectively).

### TSSM bioassays in the laboratory

To further understand and identify TSSM resistance mechanisms in tomato grafts, laboratory assays were performed on grafts onto R15391 and R312064, which had the lowest populations of TSSM movable stages, as well as ungrafted plants (control) (Table 2). Antibiosis and antixenosis resistance mechanisms to TSSM in grafts were investigated using no-choice assay and biological and behavioral bioassays. The life table parameters were estimated to provide a detailed description of a population’s development, survival, fecundity, and life expectancy.

#### Duration of TSSM developmental stages

Table 4 displays the development duration from egg to adult for TSSM males and females that feed on leaves of ungrafted plants and grafts onto R312064 and R15391. TSSM females and males developed effectively on grafted and ungrafted plant leaf discs. The developmental duration of the egg, larva, protonymph, deutonymph, and adult of both TSSM sexes differed significantly between grafted and ungrafted plants, except for the adult male (Table 4). Feeding onto leaves of grafts onto R-312064 increased the durations for the egg, larva, protonymph, deutonymph, and adult of TSSM females (4.45, 3.40, 3.20, 2.80, and 17.90 days, respectively) and males (4.33, 4.00, 3.83, 3.33, and 14.17 days, respectively) (Table 4). TSSM females and males developed faster on ungrafted plants and graft-R15391, with no significant differences (*P* < 0.05) between them (Table 4).

**Table 4:** Developmental durations (days ± standard error) of males and females^z^ *Tetranychus urticae* Koch. reared on graft-R312064, graft-R15391, and ungrafted plants of tomato ‘Nairouz F_1_’

Sex	Developmental stage	Graft-R312064^z,y^		Graft-R15391^z,y^		Ungrafting^z^	
**Female**		(20)		(22)		(24)	
	**Egg**	4.45 ± 0.11	a	4.09 ± 0.16	ab	3.92 ± 0.17	b
	**Larva**	3.40 ± 0.11	a	2.00 ± 0.13	b	2.25 ± 0.15	b
	**Protonymph**	3.20 ± 0.11	a	2.23 ± 0.13	b	2.42 ± 0.13	b
	**Deutonymph**	2.80 ± 0.15	a	2.18 ± 0.15	b	2.42 ± 0.13	b
	**Pre-adult**	13.85 ± 0.26	a	10.50 ± 0.31	b	11.00 ± 0.25	b
	**Adult longevity**	17.90 ± 0.34	a	15.77 ± 0.33	b	16.21 ± 0.30	b
	**Life span**	31.75 ± 0.47	a	26.27 ± 0.44	b	27.21 ± 0.41	b
**Male**		(6)		(5)		(4)	
	**Egg**	4.33 ± 0.21	a	3.80 ± 0.37	ab	3.25 ± 0.24	b
	**Larva**	4.00 ± 0.26	a	2.80 ± 0.37	b	2.25 ± 0.25	b
	**Protonymph**	3.83 ± 0.31	a	3.00 ± 0.36	b	2.50 ± 0.28	b
	**Deutonymph**	3.33 ± 0.21	a	2.80 ± 0.37	ab	2.25 ± 0.24	b
	**Pre-adult**	15.50 ± 0.22	a	12.40 ± 0.68	b	10.25 ± 0.47	c
	**Adult longevity**	14.17 ± 0.31	a	14.00 ± 0.31	a	14.00 ± 0.40	a
	**Life span**	29.67 ± 0.33	a	26.40 ± 0.68	b	24.25 ± 0.83	c

^z^Means value ± standard error (the number of replicates varied by grafting treatment and by sex, as shown in the parenthesis). The means in each row with the same letters are not significantly different (Paired bootstrap test, *P* ≤ 0.05)

^y^Rootstocks were interspecific hybrids between *Solanum lycopersicum* LA0815 cv. VFN-14, as a female parent, and either of *S. habrochaites* AusTRCF312064 (R-312064) or CGN15391 (R-15391), as male parents

TSSM reared at 27 ± 3 °C and 65 ± 5% RH with a photoperiod of 16L:8D

Table 4 shows significant differences (*P* < 0.05) in the total developmental duration from egg to adult (life span) for both TSSM sexes between grafted and ungrafted plants. TSSM females and males grew more slowly when fed on leaves of grafts onto R312064, with life spans of 31.75 and 29.67 days, respectively. In contrast, both sexes developed faster by feeding on leaves of ungrafted plants (life spans of 27.21 and 24.25 days, respectively) and grafts onto R15391 (life spans of 26.27 and 26.40, respectively), with no significant differences (*P* < 0.05) between them except for males (Table 4).

#### Duration of pre-oviposition and oviposition, and fecundity of TSSM females

The period of adult pre-oviposition (APOP), total pre-oviposition (TPOP), and oviposition, as well as the fecundity of TSSM females, were significantly impacted by tomato grafting, as shown in Table 5. The lowest APOP and TPOP values were observed with ungrafted plant leaf discs (10.54 and 12.54 days, respectively). The APOP and TPOP increased by feeding TSSM on leaves of grafts onto R-312064 (3.35 and 17.20 days, respectively) and R-15391 (2.14 and 12.64 days, respectively), with significant differences (*P* < 0.05) among them (Table 5). The highest oviposition period (day) was found on leaf discs of grafts onto R312064 (12.75) and ungrafted plants (12.00), with no significant differences among them, while the lowest was found on leaf discs of grafts onto R15391 (11.82). The oviposition period was responsible for 71.23, 74.41, and 74.04% of the lifespan of TSSM females, who fed on leaves of grafts onto R-312064 and R-15391, and ungrafted plants, respectively. Total fecundity (eggs female^−1^) of TSSM females decreased when fed on leaf discs of grafts onto R-312064 (21.95%) (Table 5). The total fecundity was higher on ungrafted plants (51.21%) and grafts onto R-15391 (51.41%), with no significant (*P* < 0.05) differences among them. The sex ratio of the new offspring (females total^−1^) was 76.92, 81.48, and 85.71% for TSSM females feeding on leaves of grafts onto R-312064 and R-15391, and ungrafted plants, respectively (Table 5).

**Table 5:** Reproductive period, total fecundity, and sex ratio of *Tetranychus urticae* Koch. reared on graft-R312064, graft-R15391, and ungrafted plants of tomato ‘Nairouz F_1_’

Biological aspects	Graft-R312064^z, y^		Graft-R15391^z, y^		Ungrafting^z^	
APOP (days)^x^	3.35 ± 0.11	a	2.14 ± 0.13	b	1.54 ± 0.10	c
TPOP (days)^x^	17.20 ± 0.26	a	12.64 ± 0.33	b	12.54 ± 0.26	b
Oviposition (days)	12.75 ± 0.29	a	11.82 ± 0.31	b	12.00 ± 0.34	a
Fecundity (eggs female^−1^)	21.95 ± 1.05	b	51.41 ± 2.48	a	51.21 ± 3.55	a
Oviposition days (%)^w^	71.23		74.93		74.04	
Sex ratio^v^ (%)	76.92		81.48		85.71	

^z^Means value ± standard error (n = 4). The means in each row with the same letters are not significantly different (Paired bootstrap test, *P* ≤ 0.05)

^y^Rootstocks were interspecific hybrids between *Solanum lycopersicum* LA0815 cv. VFN-14, as a female parent, and either of *S. habrochaites* AusTRCF312064 or CGN15391, as male parents

^x^*APOP*: adult preoviposition period and *TPOP*: total preoviposition period

^w^The percentage of oviposition days

^v^Sex ratio is the proportion of adult females in total offspring individuals

TSSM reared at 27 ± 3 °C and 65 ± 5% RH with a photoperiod of 16L:8D

#### Two-sex life table parameters

**Fig. 6:**
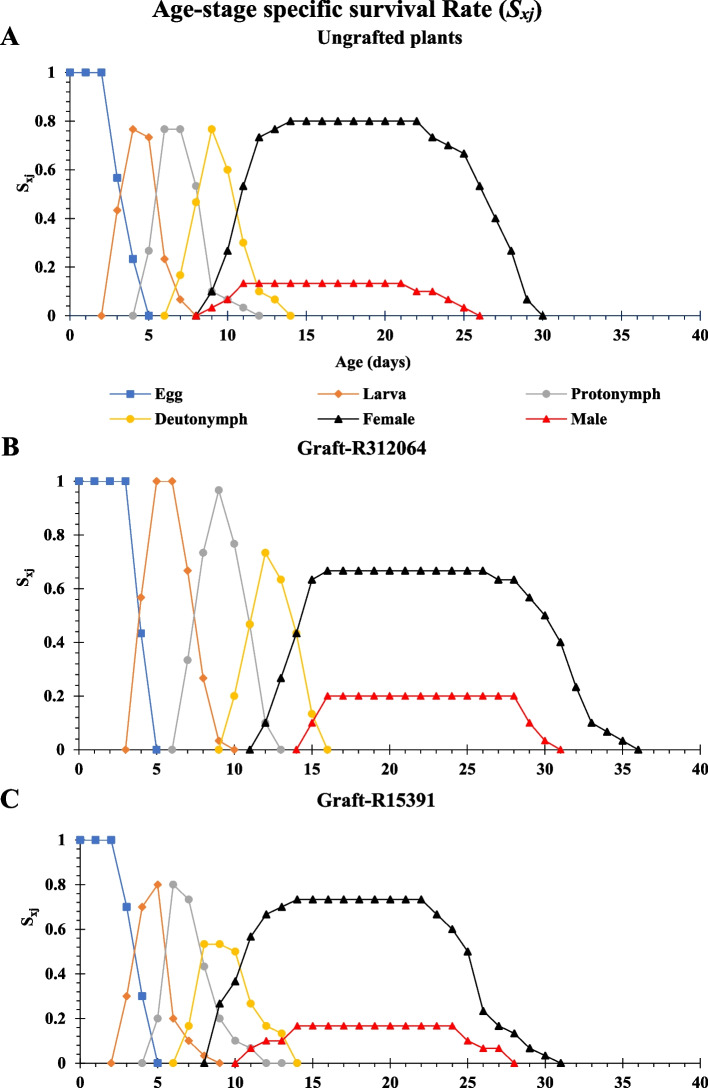
Survival rates (*S*_*xj*_) of *Tetranychus urticae* feeding on leaves of tomato ‘Nairouz F_1_’ ungrafted (**A**) and grafted plants onto R312064 (**B**) and R15391 (**C**). Rootstocks were interspecific hybrids between *Solanum lycopersicum* LA0815 cv. VFN-14, as a female parent, and either of *S. habrochaites* AusTRCF312064 (R-312064) or CGN15391 (R-15391) as male parents

The probability of a newborn surviving to age x and developing to stage j is represented by the age-stage-specific survival rates (*S*_*xj*_) of TSSM females and males, as shown in Fig. 6. The curves in Fig. 6 depict survival, variable developmental rates, and stage differentiation. Significant differences were observed among TSSM feeding on grafted and ungrafted plant leaves, with variations in *S*_*xj*_ peaks showing up for all developmental stages except for the egg. Feeding on leaves of grafts-R312064 increased *S*_*xj*_ during the larva, protonymph, and deutonymph stages (1, 0.97, and 0.73, respectively), compared to feeding on those of ungrafted plant leaves (0.77 for all stages) and grafts-R15391 (0.8, 0.8, and 0.53, respectively). The lifespan of these TSSM stages on grafted plant leaves was longer than on ungrafted plant leaves. The probability of a newborn egg surviving until the female adult stage was reduced from 0.80 with ungrafted plants to 0.73 and 0.67 with grafts onto R15391 or R312064, respectively (Fig. 6). Feeding male adults on leaves of grafts onto R-312064 or R-15391 increased *S*_*xj*_ to 0.2 and 0.16, respectively, compared to 0.13 when fed on ungrafted plant leaves (Fig. 6). The lifespan of male adults on grafted plant leaves was longer (> 25 days) than those on ungrafted plant leaves. According to these results, feeding on grafts-312064 leaves resulted in the lowest survival probability of a newborn egg to the female adult stage.

#### Age-specific survival rate and fecundity curves

**Fig. 7:**
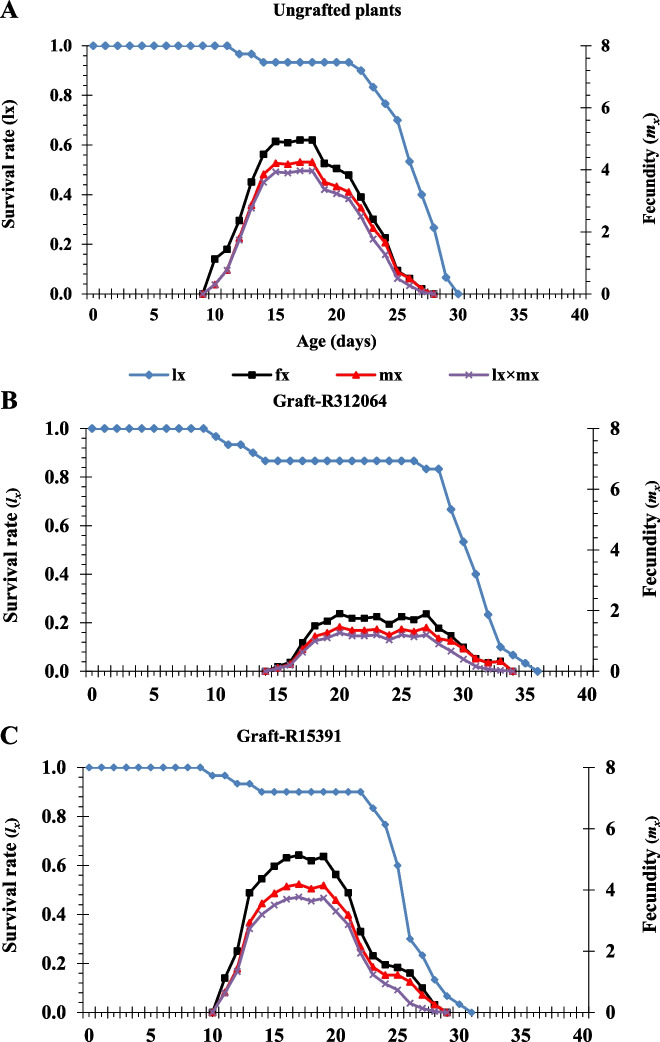
Age-specific survival rate (*l*_*x*_), age-stage fecundity (*f*_*xj*_), age-specific fecundity rate (*m*_*x*_), and age-specific maternity (*l*_*x*_*m*_*x*_) of *Tetranychus urticae* feeding on leaves of tomato ‘Nairouz F_1_’ ungrafted (**A**) and grafted plants on R312064 (**B**) and R15391 (**C**). Rootstocks were interspecific hybrids between *Solanum lycopersicum* LA0815 cv. VFN-14, as a female parent, and either of *S. habrochaites* AusTRCF312064 (R-312064) or CGN15391 (R-15391) as male parents

The age-specific survival rate (*l*_*x*_) ignoring stage differentiation (probability that an egg will survive to age x), age-stage specific fecundity (*f*_*xj*_), age-specific fecundity (*m*_*x*_), and age-specific net maternity (*l*_*x*_*m*_*x*_) for TSSM fed on leaves of grafted and ungrafted plants are plotted in Fig. 7. The *l*_*x*_ remained stable until day 11 with ungrafted plants (Fig. 7A), while it decreased after 9 days with grafted plants (Fig. 7B&C). The survival decreased rapidly on days 23, 22, and 28 with ungrafted plants (Fig. 7A), grafts-R15391 (Fig. 7C), and grafts-R312064 (Fig. 7B), respectively. The age-stage specific fecundity (*f*_*xj*_) indicates the number of eggs produced by adult females of age x, where the age x is counted from the egg stage. The *f*_*xj*_ showed that female adults began to reproduce at the age of 15, 11, and 10 days when fed on leaves of grafts-R312064 (Fig. 7B), grafts-R15391 (Fig. 7C), and ungrafted plants (Fig. 7A), respectively. The age-specific fecundity (*m*_*x*_) indicates the daily number of eggs produced by females at age x. The peak of *m*_*x*_ occurred on day 17, with 4.19 and 4.25 egg individual^−1^ day^−1^ in grafts-R15391 (Fig. 7C) and ungrafted plants (Fig. 7A), respectively. The peak *m*_*x*_ occurred on day 20, with 1.46 egg individual^−1^ day^−1^ in grafts-R312064 (Fig. 7B). The age-specific net maternity (*l*_*x*_*m*_*x*_) indicates the net fecundity of the population at age x. The *l*_*x*_*m*_*x*_ value was lower with grafts-312064, reaching a peak value of 1.27 on day 20 due to the low *l*_*x*_ (Fig. 7C). The *l*_*x*_*m*_*x*_ reached a peak value of 3.97 and 3.77 for ungrafted plants and grafts-R15391 on day 17, respectively (Fig. 7A&C).

#### Life table parameters of TSSM

The paired bootstrap test revealed significant differences (*P* < 0.05) in the life history parameters, including mean generation time (*GT*), doubling time (*DT*), net reproductive rate (*R*_*0*_), gross reproductive rate (*GRR*), intrinsic rate of increase (*r*_*m*_), and finite rate of increase (*λ*), between grafted and ungrafted plants, as shown in Table 6. The highest GT (day^−1^) and DT (day^−1^) were with grafts-R312064 (23.33 and 6.03, respectively), and the lowest were with ungrafted plants (17.30 and 3.23, respectively), which were not significantly different from those with grafts-R15391 (17.51 and 3.34, respectively; Table 6). *R*_*0*_ (offspring individual^−1^) and *GRR* (offspring individual^−1^) increased and peaked with ungrafted plants (40.97 and 44.76, respectively), but were not significantly different (*P* < 0.05) from those with grafts-R15391 (37.70 and 43.68, respectively; Table 6). *R*_*0*_ and *GRR* decreased with grafts-R312064 (14.63 and 18.23, respectively; Table 6). The highest *r*_*m*_ (day^−1^) and *λ* (day^−1^) were found with ungrafted plants (0.215 and 1.239, respectively) and grafts-R15391 (0.207 and 1.230, respectively), with no significant differences (*P* < 0.05) between them (Table 6). The lowest *r*_*m*_ and *λ* were with graft-R312064 (0.115 and 1.122, respectively).

**Table 6:** Life table parameters of *Tetranychus urticae* Koch. on graft-R312064, graft-R15391, and ungrafted plants of tomato scion ‘Nariouz F_1_’

Life table parameters	Graft-R312064^x,y^		Graft-R15391^x,y^		Ungrafting^x^	
Mean generation time (G*T*; day^−1^)	23.33 ± 0.320	a	17.51 ± 0.320	b	17.30 ± 0.340	b
The doubling time (*DT*; day^−1^)	6.03 ± 0.340	a	3.34 ± 0.130	b	3.23 ± 0.110	b
Net reproductive rate (*R*_*0*_; offspring)	14.63 ± 1.980	b	37.70 ± 4.310	a	40.97 ± 3.840	a
Gross reproductive rate (*GRR*; offspring)	18.23 ± 1.940	b	43.68 ± 4.280	a	44.76 ± 3.440	a
Intrinsic rate of increase (*r*_*m*_; day^−1^)	0.115 ± 0.006	b	0.207 ± 0.008	a	0.215 ± 0.007	a
Finite rate of increase (*λ*; day^−1^)	1.122 ± 0.007	b	1.230 ± 0.010	a	1.239 ± 0.009	a

^z^Means value ± standard error. The means in each row with the same letters are not significantly different (Paired bootstrap test, *P* < 0.05)

^y^Rootstocks were interspecific hybrids between *Solanum lycopersicum* LA0815 cv. VFN-14, as a female parent, and either of *S. habrochaites* AusTRCF312064 or CGN15391, as male parents

TSSM reared at 27 ± 3 °C and 65 ± 5% RH with a photoperiod of 16L:8D

## Discussion

Tomato grafting has become an important cultivation technique for sustainable agriculture production, helping to improve the efficiency of modern cultivars by overcoming abiotic and biotic stresses [47], and increasing plant growth and productivity [15]. This is achieved by grafting onto compatible, stress-tolerant/resistant, and strong-rooted rootstocks, which influences the performance of the scion, making it stress-tolerant/resistant and increasing the uptake and transport of water and nutrients [27]. Identifying and using the appropriate rootstocks is crucial for grafting efficacy [47]. This is the first research to investigate the efficiency of grafting tomato ‘Nairouz F_1_’ onto interspecific hybrids with *S. habrochaites* carrying the *Mi-1* gene, on TSSM resistance. Keskin and Kumral [36] found that the interspecific hybrid rootstock ‘Beaufort’ was resistant to TSSM, with significantly lower populations of TSSM. The availability of previous studies on vegetable grafting, particularly tomato, for insect control is limited. Alam et al. [21] reported that tomato grafts onto wild *Solanum* sp. had a lower population of *B. tabaci* compared to ungrafted plants. According to Alvarez-Hernandez et al. [22], six *S. lycopersicum* var. *cerasiforme* rootstocks reduced the populations of *B. tabaci*, *B. cockerelli*, and *A. gossypii* on tomato scion, while only two rootstocks had a little influence on *M. euphorbiae*. Žanić et al. [24] and Mandušić et al. [23] found that grafting tomato ‘Clarabella’ onto commercial rootstocks ‘Arnold’, ‘Buffon’, ‘Emperador’, and ‘Maxifort’ reduced the adult and nymphal populations of both *B. tabaci* and *T. vaporariorum*.

The presence and zygosity states of the *Mi-1* gene in the rootstocks and scion used in this study were initially determined using molecular markers. Several molecular markers were used to detect the *Mi-1* gene’s presence and zygosity states in tomato germplasm [37–39]. In earlier studies [39, 48], the Rex-1 marker was used. The Rex-1 marker was ineffective for *S. habrochaites* and *S. chilense* lines or hybrids, especially that harbored the *Ty-1* gene, tomato yellow leaf curl virus resistance gene. False positive findings were produced in plants containing the *Ty-1* gene by the Rex-1 marker due to the proximity of the *Mi-1* and *Ty-1* genes on chromosome 6 [37, 38]. Furthermore, the amplified Rex-1 marker products must be digested by the Taq-` restriction enzyme to identify the zygosity states of the *Mi-1* gene [39]. Therefore, new molecular markers, such as Mi23 and PMi12 markers, were developed to detect the presence of the *Mi-1* gene in *Ty-1-*positive germplasm. PMi12 and Mi23 markers are reliable, don’t need restriction digestion, and can distinguish between homozygous and heterozygous resistant genotypes [37, 38]. As a result, in later studies, including this one, the presence of the *Mi-1* gene was detected by various molecular markers, including one or both of the Mi23 or PMi12 markers, and possibly the Rex-1 marker [28, 49–51]. Despite a discrepancy in scion’s results, the three markers were consistent across all tomato rootstocks in this study. Commercial rootstocks and interspecific hybrids with *S. habrochaites* had a heterozygous gene (*Mi-1*/*mi-1*). Tomato scion 'Nairouz F_1_' showed a positive result with the Rex-1 marker, but negative results with the Mi23 and PMi12 markers. Mahmoud et al. [52] reported that 'Nairouz F_1_' harbors a *Ty-1* resistance gene, resulting in a positive Rex-1 marker test. The *Mi-1* gene is not present in ‘Nairouz F_1_’, as indicated by the negative results with the Mi23 and PMi12 markers.

The TSSM population on tomato scion ‘Nairouz F_1_’ leaves can feed, survive, and develop at various levels depending on the rootstocks (Table 2). Grafting onto interspecific hybrids with *S. habrochaites* carrying *Mi-1*/*mi-1* reduced the population of TSSM moveable stages on the grafts compared to ungrafted plants (Table 2 and Fig. 4). In the same context, Abd El-Wanis et al. [53] reported that the population of TSSM moveable stages reduced on eggplant grafts. Consistently increasing the age of tomato grafted and ungrafted plants increases the population of TSSM moveable stages (Fig. 2), which is due to the increase in plant leaf area, which increases the surface area exposed to infestation. The TSSM population on grafted and ungrafted tomato plants varied across both seasons (Table 2). The second season had a lower TSSM population than the first season (Table 2 and Figs. 3 and 4). The climate differences between them, as depicted in Fig. 1, could be the cause of this. The assessment period for the second season, from late August to early November, had higher maximum temperatures, more photosynthetic active radiation, and fewer clouds than the first one (Fig. 1). Thus, the tomato plants had higher temperatures in the second season than in the first. Riahi et al. [54] stated that TSSM activity and reproduction gradually decrease as temperatures > 30°C.

The differences in the population of TSSM moveable stages between tomato ‘Nairouz F_1_’ grafted and ungrafted plants are related to changes in plant nutritional content [27] and hormonal activity [55]. Also, grafting and rootstock alter the plant's defensive response [14, 47]. The plant’s defensive response against herbivore insects is primarily based on lowering the fitness cost of herbivore-induced injury to the plant (tolerance), reducing the pest’s preference for the plant (antixenosis resistance); or decreasing the pest’s fitness by increasing developmental time and reducing growth and survival on the plant (antibiosis resistance). Plant–insect interactions, particularly resistance, are largely influenced by phenolic compounds through various defense mechanisms. Insect antifeedants, digestibility reducers, and direct toxicants are some of the defensive effects of phenolics, which lead to antibiosis resistance. Phenolic compounds disrupt insect physiological processes, influencing insect feeding, behavior, growth, and development [56]. Phenolics responsible for antibiosis resistance are often generated at higher doses during herbivore feeding, which deters the herbivore from feeding further [2]. Furthermore, some phenolic compounds, such as gallic acid, vanillic acid, syringic acid, and chlorogenic acid, have shown repellent effects against some insects, including TSSM [56]. This phenomenon is commonly seen in nature as passive resistance, although herbivore attacks can also generate it as induced resistance. Many reports and literature indicate the significance of plant phenolics in herbivore insect resistance [56]. In this study, ungrafted plants exhibited TSSM resistance as evidenced by their leaf phenolic content of methyl gallate, coffeic acid, rutin, coumaric acid, ferulic acid, naringenin, and taxifolin that were sometimes greater than those of the grafts (Table 3). Tomato grafting onto interspecific hybrids with *S. habrochaites* carrying *Mi-1*/*mi-1* induced constitutive resistance to TSSM. Grafting tomato 'Nairouz F_1_' induced the foliar synthesis of chlorogenic acid, syringic acid, pyrocatechol, and vanillin (Table 3). Foliar gallic acid synthesis was also increased by grafting compared to ungrfated plants (Table 3). These polyphenolics showed toxicity against insect pests (antibiosis), disrupting essential physiological processes [57]. Beroza and Green [58] indicated that chlorogenic acid, syringic acid, pyrocatechol, vanillin, and gallic acid were repellents (antixenosis) to some insect species. Rani et al. [59] found that the egg parasitoid *Trichogramma chilonis* Ishil for insects showed an attraction towards these polyphenolics, stimulating biological control of herbivorous insects, including TSSM.

Chlorogenic acid (CGA: C_16_H_18_O_9_) is a crystalline phenolic compound that results from the esterification of caffeic acid and the aliphatic alcohol (–) quinic acid. CGA is a primary plant phenol component for defense response, immune regulation, and plants' biotic/abiotic stress response. CGA is a potential botanical insecticide metabolite that naturally occurs in all parts of various plants, especially in solanaceous plants [60]. CGA negatively affects the feeding behavior, growth, development, and reproduction of plant pests (antibiosis), and can even lead to the death of some insect species, including TSSM [57]. In solanaceous plants, strong anti-nutritive properties of CGA were exhibited against various herbivores, including tomato fruit worm, *Heliothis zea* [61], *Spodoptera exigua* [62], *S. litura* [63], and *Trichobaris mucorea* [64]. Elmasry et al. [65] (2020) reported that exogenous spraying of a water-alcohol extract CGA reduced the TSSM population by 100%, with the lethal concentrate (LC) 50% being 496.93 ppm and the LC 90% being 6457.47 ppm. All *S. lycopersicum* × *S. habrochaites* rootstocks carrying *Mi-1*/*mi-1* induced CGA synthesis in the tomato 'Nairouz F_1_' scion (Table 3). Indu Rani et al. [66] reported that phenolic components, including CGA, were integral to the roots of tomato RKN-resistant. D’Orso et al. [67] indicated that genes associated with chlorogenic acid biosynthesis were highly expressed in root tissues of solanaceous plants, including tomato. Therefore, a relation between the *Mi-1* gene and CGA synthesis genes can be concluded, which has to be confirmed by further genetic studies.

Syringic acid, pyrocatechol, and vanillin appeared in some grafts, particularly those onto R-1777 (Table 3). Syringic acid is a simple phenolic compound found in various crops, and it contributes to the plant’s defense against TSSM [68, 69]. Vanillin, a phenolic aldehyde, exhibits herbivore-repellent properties (antixenosis), including TSSM [70]. Kielkiewicz [68] reported that feeding of the scarlet spider mite (*T. cinnabarinus* Boisduval) on tomato leaves 'Slonka' increased the phenolics synthesis of gallic acid, vanillic acid, syringic acid, coumaric acid, ceffeic acid, ferulic acid, and chlorogenic acid.

HPLC analysis showed that grafting tomato 'Nairouz F_1_' on interspecific hybrids carrying the *Mi-1* gene induces the foliar synthesis of herbivore-repellent (antixenosis) and antibiosis phenolics, i.e., chlorogenic acid in all grafts and syringic acid, pyrocatechol, and vanillin in certain grafts. Comparative information on TSSM’s growth, survival, and reproduction on grafted and ungrafted tomato plants is unavailable. To evaluate antixenosis and antibiosis resistance in tomato grafts, TSSM males and females were reared on leaves of grafts onto R312064 and R15391, along with ungrafted plants. TSSM bio-behaviors and two-sex life table parameters were estimated to predicate future demographic changes in the TSSM population [36].

The bioassay findings (Tables 5 and 6) showed that TSSM could feed, survive, develop, and reproduce on both grafted and ungrafted tomato ‘Nairouz F_1_’ plants. Grafts-based rootstock has an impact on TSSM development and fertility. The duration of egg, larva, protonymph, deutonymph, and adult developmental stages for TSSM males and females was extended (Table 4), and the duration of adult female oviposition, pre- (APOP), and total-oviposition (TPOP) was decreased by rearing on grafts-R312064 leaves (Table 5) in comparison to rearing on grafts-R15391 and ungrafted plants. The immature development duration (day) for TSSM females was generally between 10.50 on grafts-R15391 to 13.85 on grafts-R312064, while for TSSM males was between 10.25 on ungrafted plants to 15.50 on grafts-R312064 (Table 4). The estimates of TSSM immature development duration were consistent with those of Keskin and Kumral [36], who estimated the TSSM-immaturity development duration ranged from 8.26 to 11.37 days at 25.0 ± 1 °C for tomato cultivars. The developmental duration of the TSSM on tomato plants was 10.41 days at 25.0 ± 1 °C, according to Osman et al. [71]. Conversely, Ahmed [72] reported that the immature developmental duration of TSSM on tomato cultivars ranged from 4.67 to 6.20 for males and 5.87 to 7.13 days for females under similar incubation conditions, which was less than our current findings. Nasr et al. [73] estimated that the total immature developmental duration at 25°C was 9.25 days. This could result from differ host plant quality and suitability.

The TSSM fecundity and sex ratio greatly influence the selection of resistant and susceptible plants. The sex ratio (females total^−1^) of TSSM ranged from 76.92 with grafts-312064 to 85.71 with ungrafted plants (Table 5). Consistent with the findings of Osman et al. [71], tomato cultivars showed a female-dominated sex ratio of TSSM, 78% at 25.0 ± 1 °C. This result demonstrated that, despite the general female bias in the sex ratio of TSSM, extrinsic factors, such as temperature and host plant quality and suitability, can modify the ratio. The fecundity of TSSM (eggs female^−1^) varied significantly among grafted and ungrafted plants. Total fecundity values ranged from 21.95 on grafts-R312064 to 51.41 on grafts-R15391. The fecundity of TSSM on tomato at 25 °C was 74.6 eggs [74], which is consistent with the present findings. Osman et al. [71] and Nasr et al. [73] found an egg count of 61.56 and 48.67 on tomato cultivars at 25 °C, respectively, which is less than this study. Also, Keskin and Kumral [36] observed lower fecundity of TSSM on the same host plant. Atalay and Kumral [75] reported 85.31 to 276.00 eggs on tomato at 27 ± 1 °C, which is higher than our present data. The differences in developmental durations, fecundity, and sex ratio between published data and this study could be caused by various factors, such as TSSM geographical strain, plant suitability, leaf surface traits, rearing methods, and the presence of phytochemical compounds [71]. Fernández-Muñoz et al. [76], Alba et al. [11], Keskina and Kumral [36], and de Oliveira et al. [12] reported that plants can resist TSSM attacks by prolonging their developmental time, reducing their fecundity and longevity, and/or attacking the pest’s natural enemies. This was observed when TSSM was grown in grafts-R312064 compared to ungrafted plants. Thus, grafts-R312064 were unsuitable for feeding TSSM.

The life table parameters can be trusted to assess the host plant's effects on the growth, survival, and reproduction of a TSSM population, as they show their population growth rates in the current and next generations [36]. Previous studies on TSSM population parameters relied on traditional analysis methods of the female age-specific life Table [77]. This method ignores the male population and variations in developmental rates among individuals in a population, which can lead to errors in the life table parameters. Chi and Liu [78] and Chi [79] established a theoretical model of life table analysis known as the two-sex life table, which considers the different ages, stages, and development rates of individuals of both sexes. The model has been used for life history studies of mites [35, 36, 72, 73, 80].

The age-specific survival rate curve (*l*_*x*_) of TSSM differed between grafted and ungrafted plants (Fig. 7). The survival rates of TSSM were initially high but quickly decreased at later stages, especially on grafts. The high mortality rates among adults were the cause of the decrease in survival rates [35, 36]. The curves of age-specific survival (*f*_*xj*_: probability of an egg surviving to age x), age-specific fecundity (*m*_*x*_: mean daily number of females progeny per female of age class x), and age-specific net maternity (*l*_*x*_*m*_*x*_: net fertility of the population at age x) curves showed that feeding TSSM females on graft leaves, especially those on R312064, delayed their maturation and reproduction, and reduced their fecundity (the number of eggs produced per day). The intrinsic rate of increase (*r*_*m*_), net reproductive rate (*R*_*0*_), and finite rate of increase (***λ***) are essential indicators of TSSM population dynamics. Several variables, such as development time, survivorship, and fecundity rate influence the *r*_*m*_. Thus, the *r*_*m*_ adequately describes the physiological characteristics of the insect regarding reproductive ability. This study's rm (females female-1 day-1) values ranged from 0.115 ± 0.006 to 0.215 ± 0.007. These *r*_*m*_ values are close to those estimated for TSSM reared on tomato cultivars [35, 36, 73]. Godzina et al. [35] reported that *r*_*m*_ values of TSSM on both tomato ‘Motelle’ and ‘Moneymaker’ were 0.2095 and 0.1710 females female^−1^ days^−1^, respectively during spring 2008, 0.1423 and 0.1215 females female^−1^ days^−1^, respectively, during summer 2008, and 0.1825 and 0.1836 females female^−1^ days^−1^, respectively during spring 2009. Keskin and Kumral [36] found that the *r*_*m*_ values ranged between 0.1129 and 0.2583 on seven tomato cultivars, and interspecific hybrid rootstock ‘Beaufort’ had the lowest *r*_*m*_ value. Nasr et al. [73] reported a *r*_*m*_ value of 0.65 females female^−1^ day-^1^ on tomato at the same temperature, which is higher than the present study. Vahdani et al. [80] reported that the *r*_*m*_ on ten tomato cultivars ranged between 0.090–0.1628. Grafts-R312064 had the longest population development of TSSM. Long development periods, a late reproduction peak, and high daily egg production and total fecundity. These findings indicated that grafts-R312064 were less TSSM-suitable plants.

Plant resistance to herbivorous pests has been assessed using life table parameters as performance indicators for pest populations [35, 36, 80]. In this study, TSSM life-table parameters significantly differed by grafted and ungrafted tomato ‘Nairouz F_1_’ plants (Table 6). This result suggested that the ability of TSSM to achieve a population size varied among grafted and ungrafted plants. The estimated net reproductive rate (*R*_*0*_; female offspring), which incorporates all demographic parameters, differed significantly among grafted (ranging from 14.63—37.70) and ungrafted tomato plants (40.97 ± 3.84) in this study. The significantly lower *R*_*0*_ on grafts-R312064 indicates their unsuitability for the rapid population growth and reproduction of TSSM [36]. The highest *R*_*0*_ was calculated for the TSSM developing on ungrafted plants and grafts-R15391. This suggests that these plants are highly suitable for TSSM growth and reproduction. The *R*_*0*_ in our study of TSSM at 27°C was similar to those reported by Osman et al. [71] and Nasr et al. [73], 36.49 and 21.9, respectively, on tomato cultivars at 25 °C. Keskin and Kumral [36] also found that *R*_*0*_ ranged between 5.818 to 26.105 of TSSM on seven tomato cultivars. Godzina et al. [35] reported that *R*_*0*_ values of TSSM on both tomato ‘Motelle’ and ‘Moneymaker’ were 67.78 and 35.16 offspring female^−1^, respectively during spring 2008, 43.34 and 21.83 offspring female^−1^, respectively, during summer 2008, and 58.31 and 66.09 offspring female^−1^, respectively during spring 2009. The *R*_*0*_ values of TSSM reared on ten tomato cultivars ranged from 4.69 to 13.15, according to Vahdani et al.^80^. The estimated finite rate of increase (λ; day^−1^) values for TSSM obtained from this study ranged from 1.122 to 1.239. Keskin and Kumral^36^ estimated λ to be 1.128–1.327 on seven tomato cultivars at 27 °C, and interspecific hybrid rootstock ‘Beaufort’ had the lowest λ value. Godzina et al. [35] found that the λ for TSSM on tomato "Motelle" (*Mi-1*/*Mi-1*) was greater than those on tomato "Moneymaker" (*mi-1*/*mi-1*) during the spring (1.2331 and 1.1865, respectively) and summer (1.1529 and 1.292, respectively) 2008 studies, in contrast to the spring 2009 trial (1.2002 and 1.2015, respectively).

Resistance of grafted and ungrafted plants to TSSM is indicated by both net reproduction rate (*R*_*0*_) and mean generation time (*GT*), which are summarized in intrinsic rate of increase (*r*_*m*_). The relatively lower *R*_*0*_ (14.63) on grafts-R312064 is a major factor affecting the *r*_*m*_ value on these grafts (0.115). The longest *GT* was calculated on this host plant (23.33 days), which can effectively result in a lower *r*_*m*_. Therefore, the TSSM population would likely be reduced on grafts-R312064 compared to ungrafted plants and graft-R15391. The shorter *GT* on ungrafted plants (17.30) and grafts-R15391 (17.51) has caused the *r*_*m*_ value to be the highest on these plant hosts, combined with the highest fecundity and reproductive rate on these host plants. The variations in life table parameters were probably a function of different food sources (host plants) taken up by the adults during larval and nymphal stages. The lower performance of some host cultivars may be due to the absence of primary essential nutrients for the growth and development of this mite, or the presence of secondary metabolites that directly affect potential herbivore development and fecundity [81]. The large differences in the *r*_*m*_, *R*_*0*_, *λ*, and *GT* values among tomato germplasm might be caused by plant age, chemical composition (particularly secondary compounds), leaf surface morphology, nutrient availability, moisture, and experimental status. In this study tomato 'Nairouz F_1_' was susceptible to TSSM, which was favorable to TSSM biology. Grafts 'Nairouz F_1_' onto R15391 did not significantly change this, but grafting onto R312064 did change this, and the grafts-R312064 were unfavorable to TSSM. This may be due to the amount and quality of the synthesized phenolic compounds. In contrast to the approximately 7 and 8 chemicals found in grafts-R312064 and grafts-R15391, respectively, roughly 6 compounds were found in the ungrafted plants (Table 3). The grafts differ from the ungrafted plants in that they synthesize chlorogenic acid (Table 3). Compared to the ungrafted plants, the grafts had a higher concentration of these chemicals. These substances were also linked to resistance to antibiosis and antixenosis.

## Conclusion

Tomato grafting onto interspecific hybrid rootstocks containing the *Mi-1* gene can induce antixenosis and antibiosis resistance to TSSM by synthesizing chlorogenic acid and other phenolics. Future research should investigate the biochemical pathways and genetic factors involved in this resistance. Graft-based rootstock influences TSSM resistance;, therefore, further evaluation of several interspecific hybrids and high-yielding scion cultivars is needed to select the most effective rootstocks with a variety of scion cultivars for pest resistance and high yields.

## Supplementary Information

Supplementary Material 1.

## Data Availability

No datasets were generated or analysed during the current study.

## Abbreviations

APOP: Adult pre-oviposition period
CRD: Randomized complete design
*DT*: Doubling time
EU: Experimental unit
*f*_*xj*_: Age-stage specific fecundity
*GRR*: Gross reproductive rate
*GT*: Mean generation time
HPLC: High-performance liquid chromatography
*l*_*x*_: Age-specific survival rate
*l*_*x*_*m*_*x*_: Age-specific net maternity
*m*_*x*_: Age-specific fecundity
*R*_*0*_: Net reproductive rate
RCBD: Randomized complete block design
*r*_*m*_: Intrinsic rate of increase
*S*_*xj*_: Age-stage-specific survival rates
TPOP: Total pre-oviposition period
TSSM: Two-spotted spider mite
*λ*: Finite rate of increase
