# Supplementary material for: Grafting Tomato ‘Nairouz F1’ onto Interspecific Hybrids for Induced Antibiosis and Antixenosis Resistance to Tetranychus urticae Koch via Chlorogenic Acid Synthesis
Source: BMC Plant Biol. 2025 Mar 6;25:295. doi: 10.1186/s12870-025-06257-8 (PMC11884129; doi:10.1186/s12870-025-06257-8)
Supplement: Supplementary file 1 — Supplementary Material 1. [file 12870_2025_6257_MOESM1_ESM.docx]

**Table S1. Two-sex life table parameters according to Chi et al. (2022).**

| **Two-sex life table** |  |
| --- | --- |
| *x* (day) | *x*: age |
| *N_x_* | *N_x_*: number of surviving individuals (females and males in adult stage) entering the age *x*. |
| *f_xj_* | *f_xj_*: age / stage-specific fecundity (daily number of eggs produced per female of age *x*). |
| *S_xj_* = $\frac{n_{xj}}{n_{01}}$ | *S_xj_* is the age-stage survival rate and *n_xj_* is the number of insects surviving to ag *x* and stage *j*. |
| *l_x_* = $\sum_{j=1}^{k} S_{xj}$ | *l*_x_: the age-specific survival rate, and *k*: number of stages. |
| *m_x_* (female female^-1^ day^-1^) = $\frac{\sum_{j=1}^{k} S_{xj}f_{xj}}{\sum_{j=1}^{k} S_{xj}}$ | *m*_x_: age-specific fecundity (daily number of eggs produced per individual i.e., this number is divided by all individuals (males and females) of age *x*). |
| *R_0_* = $\sum_{x=0}^{\omega} \sum_{x=1}^{k} l_{x}m_{x}$ | *R*_0_: the net reproduction rate, *x*: the age, *k*: the maximum age for each stage, *l_x_*: the age-specific survival rate, and *m_x_*: the age-specific fecundity. |
| *r* (day^-1^) = $\sum_{x=0}^{\omega} e^{-r(x+1)}l_{x}m_{x}$=1 | *r*: the intrinsic rate of increase (number of individuals added to the population per individual per day, i.e. the intrinsic birth rate (*b*) minus the intrinsic death rate (*d*)) (day^-1^) |
| *T =* $\frac{lnR_{0}}{r}$ | *T*: the mean generation time (day) |
| *λ* (day^-1^) = *e^r^* | *λ*: finite rate of increase (the rate at which the population (females and males) increases from one day to the next day; day^-1^), *r*: the intrinsic rate of increase. |
| *GRR* (female offspring) = $\sum_{x=\alpha}^{\beta} m_{x}$ | *GRR*: Gross reproduction rate (eggs per individual) |
| *r_c_* _=_ $\frac{{log}_{e}R_{0}}{T}$ | *r_c_*: growth capacity rate. |
